# Bone Morphogenetic Proteins: A Promising Approach for Enhancing Fracture Healing

**DOI:** 10.7759/cureus.66619

**Published:** 2024-08-11

**Authors:** Vinod Nair, Vishal S Patil, Amogh Todkar, Meet Shah, Shivappa Devarmani

**Affiliations:** 1 Orthopedics, Dr. D. Y. Patil Medical College, Hospital and Research Centre, Dr. D. Y. Patil Vidyapeeth (Deemed to be University), Pune, IND

**Keywords:** non-union, osteosynthesis, fracture healing, bone morphogenetic proteins, bone

## Abstract

Fracture healing is a complex biological process that can be delayed or impaired in certain situations. Bone morphogenetic proteins (BMPs) have emerged as a promising therapeutic strategy to promote bone formation and accelerate fracture healing. This editorial talks about the current understanding of BMPs, their mechanisms of action in fracture healing, and their potential applications in orthopedic trauma management. We also discuss the ongoing challenges and future directions for research on BMPs in fracture healing.

## Editorial

Fractures are a common orthopedic issue, affecting millions of people globally. While most fractures heal naturally, some cases experience delayed union, non-union, or malunion. These complications can significantly impact patients' mobility and quality of life, requiring additional interventions. Bone morphogenetic proteins (BMPs) are a class of signaling molecules that are a subfamily of transforming growth factor beta. Since the discovery of BMPs' ability to stimulate bone development, new insights into the cellular and molecular mechanisms underlying these proteins have enabled the use of the growth factor to accelerate fracture healing. They stimulate various cellular processes essential for fracture healing, leading to osteoblast differentiation, cartilage formation, and vascularization. Osteoprogenitor cells, osteoblasts, chondrocytes, and platelets all create bone-forming proteins (BMPs) in the bone [1]. BMPs are strong osteoblast-differentiation agents that stimulate osteoblast precursor cells and osteochondrogenic lineage cells to differentiate from multipotent mesenchymal cells.

This family consists of dimeric molecules, comprising a seven-cysteine TGF-beta domain in their C-termini [2]. About 20 members of the BMP family have been found and described thus far. BMP-2, BMP-6, BMP-7, and recombinant BMP-7 (rhBMP7) are now being widely used in complex orthopedic trauma conditions either as an add-on or as an alternative. Cellularly, BMPs diffuse along a concentration gradient and bind to receptors on the plasma membranes of different types of cells, including mesenchymal stem cells and osteoblasts, to induce differentiation and proliferation in a specific spatial pattern.

Initially, all BMPs are synthesized as precursor proteins that have three different peptides: a prodomain for folding and secretion, a C-terminal mature peptide, and an N-terminal signal peptide. The cytoplasm produces the precursors, which are cleaved to yield the N- and C-terminal fragments. It is possible for the mature C-terminal segment to connect to its receptor [3]. BMPs have the ability to communicate via both canonical and noncanonical routes. Regarding the canonical pathway, it has been shown that BMPs start the signal-transduction cascade by attaching themselves as ligands to cell surface receptors and thereby creating a hetero-tetrameric complex with two dimers of serine/threonine kinase receptors of type I (BMPR-I) and type II (BMPR-II). Then, intracellular effector proteins and receptor-regulated SMADs are phosphorylated by the active type I receptors, activating SMAD1, SMAD5, and SMAD8 (SMAD1/5/8) [4]. After receptor-regulated SMADs are activated, a complex with the comediator SMAD, SMAD4, is formed. This complex moves to the nucleus and acts as a transcription factor, which controls the expression of certain genes. There are other pathways described to mediate the osteoinductive signals of BMPs, in addition to the SMAD-dependent activation. These include the SMAD-independent p38 mitogen-activated protein kinase pathway and the phosphatidylinositol 3-kinase/protein kinase B (AKT) pathway, which is a noncanonical pathway [5].

Once there is capillary invasion, the chondrocytes undergo calcification and hypertrophy, and new bone formation takes place within 9-12 days. 

The role of BMPs in fracture healing is evolving. However, they have shown promising results in several clinical applications, including the treatment of non-unions and malunions by injecting directly into the fracture site to stimulate bone formation and promote healing in cases where conservative management has failed. Spinal fusion surgery is used to enhance bone fusion between vertebrae in spinal fusion procedures such as transforaminal lumbar interbody fusion (TLIF) and in tibial plateau fractures to promote healing in complex tibial plateau fractures, which are prone to delayed union and non-union.

The Food and Drug Administration (FDA) has approved recombinant BMP-2 (rhBMP2) for open tibial fractures and rhBMP7 as a device exemption for tibial non-unions. The FDA has also approved posterior and transforaminal lumbar interbody fusion and cervical fusion as well as maxillary sinus and alveolar ridge augmentation to fulfill tooth extraction sockets and intradental defects and enable the installation of dental implants.

While BMPs hold immense promise for promoting fracture healing, their successful clinical application hinges on optimal delivery techniques. The ideal delivery method should ensure targeted delivery of BMPs directly to the fracture site to maximize their therapeutic effect at the desired location. The technique should have sustained releasing properties over a prolonged period, which is crucial to maintaining sufficient therapeutic levels throughout the healing process. The method should be minimally invasive, as such techniques are preferred to minimize additional tissue disruption and potential complications.

Several techniques are currently employed for BMP delivery in fracture healing, which include direct injection of a concentrated BMP solution into the fracture gap under fluoroscopic guidance (using C-arm intraoperatively) to ensure precise placement.

Injectable hydrogels BMPs can be incorporated into injectable hydrogels that form a scaffold at the fracture site. These hydrogels provide sustained release of BMPs and may promote cell attachment and growth. Lastly, with demineralized bone matrix (DBM) carriers, DBM - a bone graft material containing natural growth factors - can be used as a carrier for BMPs. This combination leverages the osteoinductive properties of BMPs with the structural support of DBM.

Cecchi et al. have reviewed in their article the dosage of BMP-7 as 3.5 mg administered bound with 1 gm of bovine collagen granules, which act as scaffolds for osteoneogenesis [5].

Ongoing research is exploring novel delivery methods to optimize BMP therapy. These include gene therapy vectors that deliver BMP genes directly to the fracture site. Using vectors could provide a more sustained and localized release of BMPs compared to protein delivery or nanoparticle-based delivery systems that can be used to encapsulate BMPs and control their release at the fracture site, potentially reducing the risk of off-target effects.

By continuing to refine injection techniques and explore innovative delivery methods, we can maximize the therapeutic potential of BMPs in promoting effective fracture healing and improving patient outcomes.

Despite their potential benefits, using BMPs in fracture healing comes with certain challenges, like high dosage requirements. Effective doses of BMPs can be high, leading to increased production costs. Second, off-target effects and high doses of BMPs may cause unwanted effects in surrounding tissues, such as ectopic bone formation. Developing optimal delivery methods for delivering BMPs to the fracture site and ensuring their sustained release is crucial.

Following long bone implantation and spinal fusion, particularly in the cervical spine, there were several major problems, including early osteolysis, heterotopic ossification, and localized, transitory edema. Patients with distal radius fractures had more severe inflammation.

After a number of clinical investigations were retrospectively analyzed, it was proposed that the first bone resorption that was shown was temporary and that subsequent bone formation took place.

Research on BMPs in fracture healing continues to explore ways to overcome these challenges. By exploring gene therapy, delivering BMP genes directly to the fracture site could provide a more targeted and sustained release of BMPs. Combinatorial therapies, which include combining BMPs with other growth factors or biomaterials, may enhance their efficacy and reduce potential side effects. Developing safer and more cost-effective BMP derivatives is ongoing, and research is ongoing to develop new BMP variants with improved safety profiles and lower production costs.

BMPs offer a promising strategy for promoting fracture healing and accelerating recovery. BMPs can be considered worthy alternatives to bone grafting in morbid patients with bone loss due to volume constraints, open wounds, or the risk of infection in and around the graft harvesting site.

As we continue to refine BMP delivery methods and address existing limitations, BMPs have the potential to become a more widely used tool in orthopedic trauma management, improving patient outcomes and reducing healthcare costs.
